# Trend Analysis of the Mortality Rates of the Top Three Causes of Death Among Chinese Residents from 2003 to 2019

**DOI:** 10.3389/ijph.2022.1604988

**Published:** 2022-09-06

**Authors:** Zemiao Zhang, Yinhuan Hu, Sha Liu, Yeyan Zhang, Jinghan Zhou, Jiayi Li, Weilin Zhu, Zhen Qi, Vivien Xi Wu

**Affiliations:** ^1^ School of Medicine and Health Management, Tongji Medical College, Huazhong University of Science and Technology, Wuhan, China; ^2^ Shenzhen Health Development Research and Data Management Center, Shenzhen, China; ^3^ Alice Lee Centre for Nursing Studies, Yong Loo Lin School of Medicine, National University of Singapore, Singapore, Singapore

**Keywords:** mortality, China, chronic disease, malignant tumors, heart disease, cerebrovascular disease, trend analysis, joinpoint regression

## Abstract

**Objective:** To delineate the mortality trends of malignant tumors, heart disease and cerebrovascular disease in China.

**Methods:** The crude mortality from 2003 to 2019 was derived from the China Health Statistical Yearbook, and the mortality rates were analyzed through joinpoint regression supplemented by descriptive statistics and χ2 tests.

**Results:** The fitting model of age-standardized mortality due to malignant tumors showed three joinpoints. The APCs from 2003 to 2005, 2005–2008, 2008–2012 and 2012–2019 were −11.00%, 9.63%, −4.67% and −1.40%, respectively, and the AAPC was −1.54%. The mortality rate of cerebrovascular disease consistently decreased (APC = AAPC = −0.98%). In the subgroup analyses, significant differences were observed between sexes and regions. The mortality rate of heart disease among rural females exhibited an upward trend (APC = AAPC = 2.33%). Older adults aged over 75 years had the highest mortality rates and the most drastic change.

**Conclusion:** The three diseases had variable change trends. The government should focus more on policies that promote the equalization of basic public health services. Continuous education on heart disease, which includes not only beneficial behaviors but also knowledge of first aid, should be strengthened for rural females.

## Introduction

Population death data are included in residential health records and are a fundamental component of the health information system. The analysis of the distribution of causes of death and the change trend of mortality rates reflects the medical service level of a country, and these data can guide the formulation of health policies and the evaluation of the effect of previous measures [1]. In terms of international practices, epidemiologists divide the disease burden into three key categories: communicable, maternal, neonatal and nutritional diseases; noncommunicable diseases (NCDs); and injuries. With the acceleration of population aging and changes in living behaviors, NCDs and their burden have attracted increasing attention [2].

In 2019, NCDs accounted for approximately 74% of global deaths [3]. Coronavirus disease 2019 (COVID-19) research has shown that patients with NCDs have a higher fatality rate [4]. In the United States, nearly half of adults are diagnosed with NCDs [5]. Premature death due to NCDs is also prominent in low- and middle-income countries [6]. Recent Chinese official statistics have demonstrated that malignant tumors, heart disease and cerebrovascular disease are the most severe chronic diseases with the highest mortality rates in China. According to global cancer statistics, the number of new cancer cases was approximately 19.3 million in 2020, with China accounting for 23.7% [7]. Regarding cerebrovascular diseases, the mortality rates in China are 4–5 times higher than those in America and Europe. Compared with Asian neighbors, this figure is much higher than that in Japan, Singapore, Thailand and other Asian countries [8].

China has been addressing NCDs for nearly 70 years, with a shift from disease-oriented to risk factor-centered strategies, and has recently focused on societal and policy measures [9, 10]. In 2009, China reformed the health system by expanding the scope of medical insurance and promoting the equalization of basic public health services. After that, policy documents such as the Regulations on National Fitness (2009), National Standards for the Prevention and Control of Chronic Diseases (Trial) (2011), China Food and Nutrition Development Program (2014–2020), and “Healthy China 2030” Planning Outline, China’s Mid- and Long-term Plan for Chronic Disease Prevention and Control (2017–2025) were successively issued [11]. It is clear that these strategies and measures have had a certain impact on the control of chronic diseases.

However, research on the epidemic status of malignant tumors, heart diseases and cerebrovascular diseases in China is insufficient. Related studies were completed early and lack comparison data and thus are not conducive to guiding the management of NCDs. Many studies are limited to certain areas or specific populations, and there are few nationwide data. Zhou et al. performed mortality comparisons and χ2 tests to analyze the characteristics of tumors, cerebrovascular diseases, heart diseases, digestive system diseases, endocrine diseases and metabolic diseases in Qinghai Province [12]. The mortality rates and trends of chronic diseases by sex and region among older adults in China were demonstrated in a study by Xia [13]. According to Wan’s analysis, the mortality of ischemic heart disease rose at an average rate of 5.08% from 1991 to 2009 due to increases in the prevalence of overweight and hyperlipidemia [14]. In Northeast China, circulatory diseases have always ranked as the leading cause of death due to the cold climate and air pollution [15, 16]. In contrast, in Sichuan and Chongqing, Western China, where people are accustomed to eating hot pot and pickled cabbage, malignant tumors are the most common cause of death [17]. Accordingly, the situation in a particular region or population cannot be generalized to the entire country; hence, we need to carry out research across various levels.

Regarding research methods, domestic studies usually apply traditional statistics to describe the distributions of diseases. Joinpoint regression is a relatively advanced method that divides changes in long time series data into several sections, which can provide more details for trend analysis [18]. Moryson et al. conducted a comparative study among 35 countries associated with the Organization for Economic Cooperation and Development (OECD) on male lung cancer between 2002 and 2017 and reported that the highest rate of decline was observed in Spain (4.9% per year), followed by Belgium (4.7% per year) and Slovakia (4.4% per year) owing to reductions in tobacco consumption [19]. Through joinpoint regression analysis, another study examined trends in heart disease mortality among adults in Mississippi and confirmed that this trend varied among subgroups (race and sex) [20]. The mortality trends of the main NCDs in Lithuania were highlighted in a study by Tamosiunas et al., who found significant downward trends from 2007 to 2008 for cardiovascular disease, coronary heart disease and malignant neoplasms by joinpoint regression analysis [21].

Accordingly, to close the research gap, this study analyzes three major chronic diseases in China (malignant tumors, heart disease and cerebrovascular disease) to delineate mortality trends by sex, region and age over a 17-year study period using joinpoint regression analysis; we hope that the Chinese experience will help guide the prevention and control of chronic diseases worldwide.

## Methods

### Data Sources

Data on mortality rates were extracted from China’s Health Statistical Yearbook (CHSYB), published by the National Health Commission (named the Ministry of Health before 2013 and National Health and Family Planning Commission between 2013 to 2018), which is based on China’s Ministry of Health Vital Registration (MOH-VR) system. The data in this system are regularly submitted to the World Health Organization (WHO) as China’s official mortality figures [22, 23]. Causes of death are coded using the International Classification of Diseases, 10th revision (ICD-10), which was issued in 1999 [24]. Malignant tumors (ICD-10 codes: C00-C97; D00-D48) comprise nasopharyngeal carcinoma, esophageal cancer, gastric cancer, colorectal and anal cancer, liver cancer, gallbladder cancer, pancreatic cancer, lung cancer, breast cancer, cervical cancer, ovarian cancer, prostate cancer, bladder cancer, brain and nervous system cancer, leukemia, and other forms of malignant tumors. Heart disease (ICD-10 codes: I05-I09; I11; I20-I27; I30-I52) includes chronic rheumatic heart disease, hypertensive heart disease, coronary heart disease, acute myocardial infarction and other heart diseases. Cerebrovascular disease (ICD-10 codes: I60-I69) includes cerebral hemorrhage, cerebral infarction, stroke, and other cerebrovascular diseases. We collected data from 2003 to 2019, for a total of 17 years. All the rates were collected and calculated as per 100,000 persons per year.

### Data Analysis

#### Mortality Standardization

In China, urban and rural areas are classified according to administrative divisions. In the MOH-VR system, cities are classified as urban areas, and counties and towns are considered rural areas. The CHSYB presents rural and urban mortality rates by sex and five-year age groups (18 age groups in total). We chose the Sixth National Population Census (NPC) of 2010 as the benchmark for the standardization of national mortality rates using the direct method. This was not only the latest population census but also near the middle of the research period. In addition, we calculated age-adjusted mortality rates in sex-, urban/rural-, and age-specific cohorts (0–19, 20–49, 50–64, 65–74 and 75 years and above). It should be noted that the scopes of rural and urban areas are different between the MOH-VR system and NPC datasets. Counties are viewed as rural areas in the MOH-VR system but are categorized as urban areas in the NPC datasets. Correspondingly, we calculated the rural mortality rates from the MOH-VR system for the combined populations of towns and counties from the NPC data and calculated the urban mortality rates for city populations according to previous studies [23, 25]. Microsoft Excel 2016 was used to organize the data and perform the calculations.

#### Joinpoint Regression Analysis

We chose the joinpoint, or segmented linear regression, model to delineate the change trends in mortality rates. We computed joinpoints using Joinpoint trend analysis software version 4.8.0.1 from the Surveillance Research Program of the National Cancer Institute (Statistical Research and Applications Branch, National Cancer Institute, US). In brief, the software uses a grid search method to analyze temporal trends in mortality rates and determines whether rate changes can be best described by a straight line (0 joinpoints) or by 1 or more linear segments (changes in slope), indicating a significant change rate [26, 27]. The use of a natural log-linear model enables the analysis of population-based disease morbidity and mortality. When the value of the dependent variable is 17–21, the recommended maximum number of joinpoints is 3. The software reports the best model to explain mortality rate trends according to the sum of squares error (SSE) and mean squared error (MSE) of each case. Tests of significance (α= 0.05) for rate changes were conducted using the Monte Carlo permutation method (4499 permutations), with Bonferroni correction for multiple testing [28]. The trend was expressed as the annual percent change (APC) of each line segment and the average annual percent change (AAPC) over the entire period. When no joinpoint was detected, the AAPC coincided with the APC [23].

#### Differences Test

In addition, we calculated the male-to-female (M/F) ratios and rural-to-urban (R/U) ratios for age-standardized mortality rates. The χ2 test, performed in SPSS 23.0 (SPSS, Chicago, IL), was used to assess the differences in mortality between sexes and regions.

## Results

### General Findings

**FIGURE 1:**
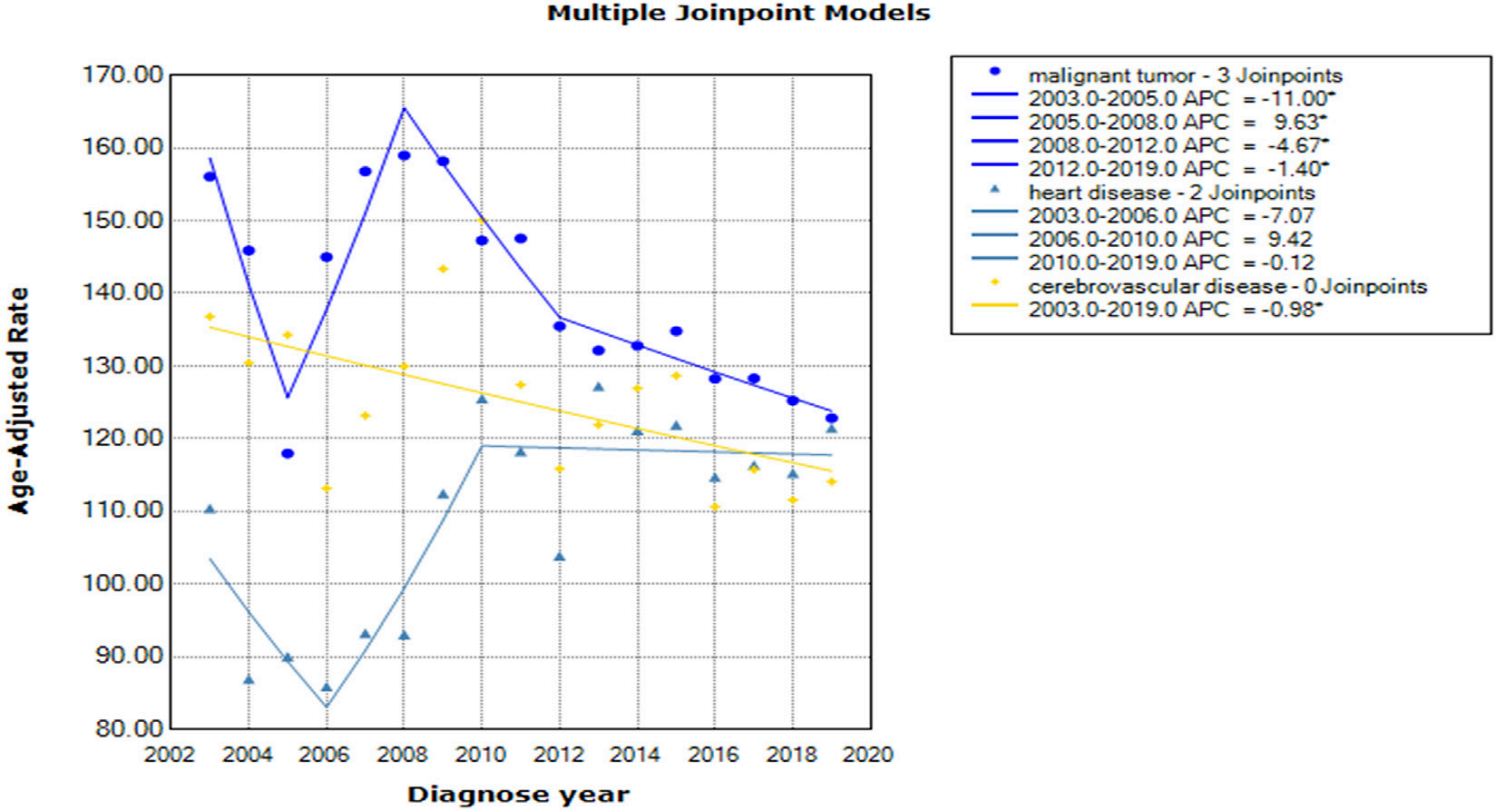
Mortality trends of malignant tumors, heart disease and cerebrovascular disease (China, 2022).

As shown in Table 1, malignant tumors were associated with the highest mortality rates during the study period, ranging from 117.97 to 156.07 per 100,000 persons. As shown in Figure 1 and Table 2, the fitting model of malignant tumors has three joinpoints; the mortality rates initially decreased sharply by 11.00%, increased by 9.63% from 2005 to 2008, and then decreased by 4.67% and 1.40% from 2008 to 2012 and from 2012 to 2019, respectively. The AAPC was −1.54% (95% CI: −2.93 to −0.12), which was statistically significant. The estimation equation was $y=e^{238.60-0.12x+0.21{\left(x-2005\right)}^{+}-0.14{\left(x-2008\right)}^{+}+0.03{\left(x-2012\right)}^{+}}$ (SSE = 0.01374, MSE = 0.00153), and the predicted rates in 2020, 2021 and 2022 were 122.04, 120.33 and 118.64 per 100,000 persons, respectively. For heart disease, the mortality rate fluctuated greatly, with a rapid decrease of 7.07% from 2003 to 2006, followed by a steep increase of 9.42% from 2006 to 2010 and then a gradual decrease of 0.12% from 2010 to 2019. However, these changes were not statistically significant. Hence, the prediction for heart disease was inconclusive. For cerebrovascular disease, no joinpoints were identified during the 17-year period. The mortality rate consecutively decreased (APC = AAPC = −0.98%, 95% CI: −1.79 to −0.17). The regression equation was $y=e^{24.70-0.01x}$ (SSE = 0.08916, MSE = 0.00594), and the predicted mortality rates in 2020, 2021 and 2022 were 114.37, 113.25 and 112.13 per 100,000 persons, respectively, showing a downward trend in the short term.

**TABLE 1:** Age-standardized mortality rates of malignant tumors, heart disease and cerebrovascular disease (China, 2022).

year	Age-standardized mortality rates (per 100,000 persons)		
	Malignant tumors	Heart disease	Cerebrovascular disease
2003	156.07	110.40	136.81
2004	145.90	86.92	130.43
2005	117.97	90.02	134.27
2006	145.00	85.90	113.18
2007	156.79	93.23	123.17
2008	158.98	93.07	129.95
2009	158.19	112.44	143.38
2010	147.28	125.52	150.11
2011	147.57	118.26	127.42
2012	135.48	103.88	115.86
2013	132.15	127.23	121.91
2014	132.80	121.16	126.93
2015	134.81	121.90	128.68
2016	128.26	114.73	110.62
2017	128.30	116.39	115.75
2018	125.24	115.28	111.55
2019	122.83	121.46	114.08

*Age-adjusted mortality rates were calculated using the 2010 standard population.

**TABLE 2:** Mortality trends of malignant tumors, heart disease and cerebrovascular disease (China, 2022).

	Mortality								
	AAPC(%) (95% CI)	Line segment 1		Line segment 2		Line segment 3		Line segment 4	
		Year	APC (%)	Year	APC (%)	Year	APC (%)	Year	APC (%)
Malignant tumors	−1.54* (−2.93, −0.12)	2003–2005	−11.00*	2005–2008	9.63*	2008–2012	−4.67*	2012–2019	−1.40*
Heart disease	0.81 (−2.35, 4.08)	2003–2006	−7.07	2006–2010	9.42	2010–2019	−0.12		
Cerebrovascular disease	−0.98* (−1.79, −0.17)	2003–2019	−0.98*						

*p < 0.05; APC, annual percentage changes; AAPC, average annual percentage changes.

### Trends in Mortality Rates by Sex

The average sex ratios (M/F) for mortality due to malignant tumors, heart disease and cerebrovascular disease were 1.78, 1.07 and 1.22, respectively, during the study period. Except for a few years, the mortality rates among males were higher than those among females. There were significant differences in mortality between sexes, and the χ2 values for the three diseases were 41.05, 5.28, and 372.19, respectively. The fitting model of mortality rates for malignant tumors among males had two joinpoints, indicating a rapid decrease from 2003 to 2005 (APC = −11.82%), followed by a rapid increase from 2005 to 2008 (APC = −9.81%) and then a gradual decrease from 2008 to 2019 (APC = -2.79%). For females, the change trend was similar to that of the overall mortality rate of malignant tumors, with three joinpoints. However, only the first period was statistically significant (APC = −8.67%). For heart disease, the gap in the mortality rate between males and females was smallest among the three conditions. The change trend was similar, with two cutoff points. However, the first joinpoint for male mortality was earlier (2003–2005) than that for female mortality (2003–2006). The AAPCs in mortality for males and females were 0.74% and 0.60%, respectively, without statistical significance. No joinpoints were identified for cerebrovascular disease among males or females throughout the period. However, females had a significant downward trend (APC = AAPC = −1.19%, 95% CI: −2.02 to −0.35). These changes are presented in Table 3 and Supplementary Figure S1.

**TABLE 3:** Mortality trends of three diseases by sex (China, 2022).

Disease	Gender	Joinpoints	AAPC (%) (95% CI)	Time segment	APC (%)	Sex ratios (M/F)	χ2 test
Malignant tumors	Male	2	−1.74*(−3.44, −0.02)	2003–2005	−11.82*	1.78	41.05*
				2005–2008	9.81*		
				2008–2019	−2.79*		
	Female	3	−1.54 (−3.28, 0.24)	2003–2005	−8.67*		
				2005–2008	5.71		
				2008–2012	−3.89		
				2012–2019	−1.05		
Heart disease	Male	2	0.74 (−2.33, 3.92)	2003–2005	−12.36	1.07	5.28*
				2005–2010	8.85*		
				2010–2019	−0.46		
	Female	2	0.60 (−2.57, 3.88)	2003–2006	−8.44		
				2006–2010	8.38		
				2010–2019	0.43		
Cerebrovascular disease	Male	0	−0.81 (−1.70, 0.08)	2003–2019	−0.81	1.22	372.19*
	Female	0	−1.19*(−2.02, −0.35)	2003–2019	−1.19*		

*p < 0.05; APC, annual percentage changes; AAPC, average annual percentage change.

### Trends in Mortality Rates by Sex and Region

There was a large difference between urban and rural areas in the mortality rates of the three conditions among both males and females. For malignant tumors, the mortality rates among urban males, rural males, urban females and rural females were 157.44, 186.50, 90.24 and 103.80 per 100,000 persons, respectively. Male and female mortality in urban areas exhibited a monotonic downward trend, without a cutoff point (male APC: −1.06%; female APC: −0.94%), whereas mortality in rural areas exhibited complex changes. The mortality rate among rural males markedly decreased from 2003 to 2005 (APC = −17.58%), increased rapidly from 2005 to 2008 (APC = 15.02%), and then decreased gradually after 2008 (APC = −3.34%). Trends in rural female mortality displayed three joinpoints, with significant decreases in the first and third periods.

For heart disease, the mortality rates among urban and rural males were 101.61 and 118.42 per 100,000 persons, respectively (χ2 = 22.03, *p* < 0.05). The change trend of mortality among urban males remained fairly stable (APC = AAPC = 0.59%, 95% CI: −0.56–1.76). The trend among rural males was characterized by two joinpoints, with a significant increase in the second section (APC = 15.19%). The mortality rates among urban and rural females were 87.25 and 112.77 per 100,000 persons, respectively, with a significant difference (χ2 = 55.19, *p* < 0.05). It is worth noting that the mortality rate among rural females presented a consecutive upward trend (APC = AAPC = 2.33%, 95% CI: 1.10–3.57). The mortality of heart disease among urban females showed no significant change (APC = 0.31, *p* > 0.05).

For cerebrovascular disease, the urban and rural mortality rates among males were 103.37 and 152.45 per 100,000 persons, respectively, while those among females were 80.23 and 127.10 per 100,000 persons, respectively. Mortality rates among males and females in rural areas were variable. Mortality trends among rural males had two joinpoints, with a gradual decrease (APC = -2.90%) in the most recent period. For mortality trends among urban and rural females, there were significant decreases of 1.21% and 1.16% per year, respectively, with no cutoff points identified. The summarized data are presented in Table 4.

**TABLE 4:** Mortality trends of three diseases by sex and region (China, 2022).

Disease	Gender	Region	Average mortality rates (per 100,000 persons)	χ2 test	Joinpoints	AAPC (%) (95%CI)	Time segment	APC (%)
Malignant tumors	Male	Urban	157.40	42.45*	0	−1.06* (−1.54, −0.58)	2003–2019	−1.06*
		Rural	186.50		2	-2.11 (−4.71, 0.57)	2003–2005	−17.58*
							2005–2008	15.02*
							2008–2019	−3.34*
	Female	Urban	90.24	15.90*	0	−0.94*(−1.44, −0.44)	2003–2019	−0.94*
		Rural	103.80		3	−1.72 (−3.60, 0.20)	2003–2005	−10.71*
							2005–2008	7.08
							2008–2012	−4.73*
							2012–2019	−0.88
Heart disease	Male	Urban	101.61	22.03*	0	0.59 (−0.56, 1.76)	2003–2019	0.59
		Rural	118.42		2	1.05 (−2.53, 4.77)	2003–2005	-17.23
							2005–2009	15.19*
							2009–2019	-0.20
	Female	Urban	87.25	55.19*	0	0.31 (-1.10, 1.74)	2003–2019	0.31
		Rural	112.77		0	2.33*(1.10, 3.57)	2003–2019	2.33*
Cerebrovascular disease	Male	Urban	103.37	159.82*	0	−0.48 (−2.25, 1.33)	2003–2019	−0.48
		Rural	152.45		2	−1.55 (−5.59, 2.67)	2003–2005	−9.46
							2005–2009	−.26
							2009–2019	−2.90*
	Female	Urban	80.23	179.30*	0	−1.21*(−2.41, 0.00)	2003–2019	−1.21*
		Rural	127.10		0	−1.16*(−2.08, −0.23)	2003–2019	−1.16*

*p < 0.05; APC, annual percentage changes; AAPC, average annual percentage change.

### Trends in Mortality Rates by Sex, Region, and Age

Table 5 shows that the age-standardized mortality rates of malignant tumors, heart disease and cerebrovascular disease increased with age (also seen in Supplementary Figure S2). The age group ≥75 years had the highest mortality rate with the most rapid change. The mortality rates of the three conditions among those aged over 75 years were 1.87, 5.08 and 4.07 times higher than those in the 65–74 age group and 4.93, 23.89 and 18.40 times higher than those in the 50–64 age group, respectively. Notably, the APCs in the mortality rates of heart disease among urban females aged over 75 years, rural females aged 65–74 years, and rural females aged over 75 years were 1.54%, 1.50% and 3.32%, respectively, without joinpoints, suggesting continuous upward trends. Another significant finding is that the mortality rates of the three conditions decreased significantly in most of the 20–49 age groups. For example, the APC values of malignant tumors, heart disease and cerebrovascular disease among urban females were -3.35%, -3.33%, and −4.21%, respectively, showing a downward trend.

**TABLE 5:** Mortality trends of three diseases by gender, region and age (China, 2022).

Disease	Region, gender	Age group	Mortality							
			Line segment 1		Line segment 2		Line segment 3		Line segment 4	
			Year	APC (%)	Year	APC (%)	Year	APC (%)	Year	APC (%)
Malignant tumors	Urban Male	0–19	2003–2019	−1.42*						
		20–49	2003–2009	−7.16*	2009–2012	10.14	2012–2019	−9.91*		
		50–64	2003–2019	−0.60						
		65–74	2003–2019	−0.40						
		≥75	2003–2006	9.73	2006–2019	−1.93*				
	Rural Male	0–19	2003–2019	−0.53						
		20–49	2003–2005	−26.19*	2005–2008	13.38	2008–2019	−4.66*		
		50–64	2003–2019	−1.71*						
		65–74	2003–2019	−0.33						
		≥75	2003–2005	−16.52	2005–2008	22.96	2008–2019	-3.80*		
	Urban Female	0–19	2003–2019	−1.34						
		20–49	2003–2019	−3.35*						
		50–64	2003–2019	−1.15*						
		65–74	2003–2019	−1.36*						
		≥75	2003–2007	7.43*	2007–2019	−0.84				
	Rural Female	0–19	2003–2019	−0.27						
		20–49	2003–2005	−17.10*	2005–2009	2.24	2009–2019	−3.87*		
		50–64	2003–2019	−2.32*						
		65–74	2003–2019	−0.78*						
		≥75	2003–2005	−8.99	2005–2008	12.07	2008–2017	−3.05*	2017–2019	7.25
Heart disease	Urban Male	0–19	2003–2005	32.92	2005–2017	−5.68*	2017–2019	11.65		
		20–49	2003–2014	3.49*	2014–2019	−4.12				
		50–64	2003–2019	2.03*						
		65–74	2003–2019	−0.84						
		≥75	2003–2019	0.57						
	Rural Male	0–19	2003–2019	−0.50						
		20–49	2003–2005	−20.07	2005–2013	9.85*	2013–2019	0.67		
		50–64	2003–2019	3.23*						
		65–74	2003–2005	−21.08	2005–2009	11.91	2009–2019	0.09		
		≥75	2003–2005	−13.30	2005–2009	17.69*	2009–2019	-1.53		
	Urban Female	0–19	2003–2006	17.94	2006–2019	−5.87*				
		20–49	2003–2019	−3.33*						
		50–64	2003–2007	−12.96*	2007–2019	0.71				
		65–74	2003–2019	−2.25*						
		≥75	2003–2019	1.54*						
	Rural Female	0–19	2003–2019	−1.71						
		20–49	2003–2005	−16.46	2005–2019	−1.12*				
		50–64	2003–2019	−0.59						
		65–74	2003–2019	1.50*						
		≥75	2003–2019	3.32*						
Cerebrovascular disease	Urban Male	0–19	2003–2019	−2.96						
		20–49	2003–2015	1.81	2015–2019	−9.38				
		50–64	2003–2019	−0.01						
		65–74	2003–2019	−1.05						
		≥75	2003–2019	−0.41						
	Rural Male	0–19	2003–2019	−0.95						
		20–49	2003–2005	−19.46*	2005–2008	12.84	2008–2014	3.85	2014–2019	-1.96
		50–64	2003–2019	−0.53						
		65–74	2003–2019	−0.84						
		≥75	2003–2009	4.81	2009–2019	−4.37*				
	Urban Female	0–19	2003–2019	−2.56						
		20–49	2003–2019	−4.21*						
		50–64	2003–2019	−3.34*						
		65–74	2003–2007	−12.56*	2007–2019	−0.65				
		≥75	2003–2019	−0.02						
	Rural Female	0–19	2003–2019	−4.28*						
		20–49	2003–2006	−14.00*	2006–2010	3.71	2010–2019	−4.60*		
		50–64	2003–2019	−3.21*						
		65–74	2003–2019	−1.09						
		≥75	2003–2019	-0.57						

*p < 0.05; APC, annual percentage changes.

## Discussion

NCDs, such as circulatory disturbance and cancers, impose a heavy burden that affects population health. This study examined the time trends of three NCDs with the highest mortality rates in China from 2003 to 2019. Despite many similar contributing risk factors, the age-standardized mortality rate of cerebrovascular disease demonstrated a downward trend over the study period, whereas malignant tumors and heart disease displayed fluctuating trends across the years.

For malignant tumors, the mortality rates initially decreased (2003–2005), steeply increased (2005–2008), and then declined steadily after 2008 among the general population and many subgroups. The increase after 2005 was largely due to a remarkable increase in mortality of gastrointestinal (GI) cancer [29]. Statistics showed that the crude mortality rates of esophageal cancer, gastric cancer, and rectal and anal cancer increased by 11.40, 7.28 and 1.54 per 100,000 persons, respectively, in rural areas during this period [29]. GI cancer is one of the most commonly diagnosed malignancies in China, particularly in the western region. Its occurrence is usually influenced by dietary habits and composition [31]. Owing to improvements in living standards and poor health literacy, a high-fat, high-protein and low-fiber diet have become popular in daily life, which has led to an increased prevalence of GI cancer. Gradually, the public has realized that only with good eating habits and diet can they keep diseases at bay. There was a notable decrease in the mortality rates of malignant tumors after 2008 associated with the implementation of the universal public health insurance system. By 2008, the New Cooperative Medical Scheme (NCMS) had basically achieved full coverage, covering 814 million or 91.5% of rural residents. This enhanced medical insurance system has achieved early detection of cancer through screening, reduced the burden of illness and encouraged health promotion among farmers [32]. In cities, the Chinese government initiated the Urban Resident Basic Medical Insurance (URBMI) in 2007 and expanded the program to 229 cities (approximately 50% of China’s cities) in 2008 [33]. Health has been identified as a top social priority by the government, making it easier for patients with chronic diseases to access timely treatment regardless of cost [34].

As the mortality rate of heart disease fluctuated greatly in this study, several regression analyses showed nonsignificant results. However, the annual mortality rates among rural females increased by 1.50% and 3.32% in the 65–74 and ≥75 age groups, respectively, suggesting deteriorating health outcomes in the aging population. Similar trends were found in Qin’s investigation of citizens in Qingdao (an urban city in China), which highlighted that the situation over the next 15 years is alarming [35]. Rural women may lack knowledge of health behaviors that target the prevention of heart disease. Moreover, they may not be aware of or trained in first aid measures such as cardiopulmonary resuscitation when in an emergency situation. It has been estimated that the number of deaths due to heart disease has increased to more than 2 million worldwide since the beginning of the twenty-first century, with more than half of the new cases occurring in the western Pacific region [36]. The reversal of this tendency through targeted actions in some Western countries is promising. Through the use of statins and hypertension prevention measures, the UK has observed a decrease in the number of deaths due to heart disease [37]. In Portugal, the close monitoring of risk factors and treatment based on medical evidence were beneficial for the control of heart disease [38]. At the end of 2011, the Department of Health and Human Services in the United States launched the Million Hearts campaign, which aimed to achieve a steady decline in heart disease-related morbidity and mortality. Targeted measures include reducing salt and trans fatty acid intake, managing blood pressure and cholesterol, banning smoking in public places, and taking aspirin when necessary; these measures are worth considering in other countries [39, 40].

According to the results, significant differences between sexes and regions, which have been identified by other scholars, were observed. When conducting an analysis of malignant tumors in the Yangpu District of Shanghai, Li noted that the male mortality rate was higher than the female mortality rate, with an average ratio of 1.45:1 [41]. From a global perspective, the sex ratios of mortality due to malignant tumors in the United States, Japan, Canada, Australia, Switzerland and Denmark were 1.29, 1.75, 1.28, 1.45, 1.47, and 1.30, respectively. Regarding cardiovascular disease, the mortality rates among males were 1.56, 1.76, 1.62, 1.51, and 1.48 times higher than those among females in the abovementioned countries [42]. In our study, especially for cerebrovascular disease, mortality rates among females showed a steady downward trend in the overall population and many subgroups, whereas mortality rates among males exhibited fluctuating changes. Usually, males have a higher exposure to risk factors such as smoking, alcohol consumption, staying up late, lack of exercise, and excessive family and work pressure, so prevention policies for chronic diseases should be targeted at the male population [43]. The distributions of most diseases have certain geographical characteristics [44]. Generally, among both males and females, mortality rates in rural areas were higher than those in urban regions, suggesting poor health awareness and behaviors among rural residents. Notably, rural mortality displayed a more complicated trend of change in this study, which may be related to the disparity in the distribution of health resources between rural and urban areas [45, 46]. Policy-makers should review health care resources in rural regions and promote the equalization of public health services.

With increasing age, physical health status declines rapidly [47]. At present, the heaviest burden of chronic diseases occurs among people over 75 years of age, and this burden has become prominent due to population aging. Similar results were reported in surveys in other provinces, such as Liaoning and Jiangsu [48, 49]. In contrast, there was a notable decrease in the age-standardized mortality rates of the three diseases in the 20–49 age group. A possible explanation for this decrease may be due to effective health education and medical treatment improvements, although further specific analyses are needed.

The AAPCs in the mortality rates of malignant tumors, heart disease and cerebrovascular disease indicated that control and treatment measures for NCDs in China have made considerable progress. Normally, the occurrence of NCDs depends on the joint action of individual genes and the external environment [50]. Prior studies indicated that the decreases in NCD mortality rates were related to multiple socioeconomic changes and personal behavioral adjustments, such as economic development, rapid urbanization, improved medical facilities, improved air quality, effective control of tobacco use, promotion of healthy diets and regular exercise [51, 52]. An increase in the health-related quality of life index can be seen not only at the country level but also at the local level. Taking Beijing as an example, in 2018, the health literacy of local residents was 32.3%, representing increases of 15.4% from 2015 to 30.8% from 2012. The sales volume of cigarette commodities was approximately 93 billion, representing a decline of 10.1% from 2014. A total of 773 sports venues were built, and 25000 fitness activities were carried out. These efforts made a positive contribution to the decrease in mortality due to NCDs, representing a decline of 0.93% from that in 2017 [53].

However, compared with other countries, the management level of chronic diseases in China is relatively low, and the control of risk factors is urgently needed. NCDs have been a top priority for the western Pacific region. Universal health coverage (UHC) is tracked with the essential health service coverage index and the proportion of the population with large out-of-pocket expenditures on health care. A UHC index indicates better accessibility and fairness of health services in a country [54]. A previous report showed that the UHC index was 83 in Japan, 86 in Singapore, 87 in Australia, and only 79 in China [36]. The alcohol consumption index was 7 in China. In contrast, this figure was only 2 in Singapore. A distinct difference in the prevalence of tobacco use can also be observed. The related rate was 24.7 in China, whereas it was 14.8, 16.2, 16.5 and 21.9 in New Zealand, Australia, Singapore and Japan, respectively [36]. This situation highlights the need for further inquiry, detailed studies and the planning of health care initiatives [55].

This study has some limitations that should be noted. First, the data on disease mortality are based on the MOH-VR system. This system contains fewer observation points from Western China, and in some cases, data are missing, which may lead to inaccurate statistical conclusions. Nevertheless, the MOH-VR system provides valuable mortality data for China via the large sample size and effective quality control measures and is widely used in research [56]. Second, malignant tumors, heart disease, and cerebrovascular disease are major categories of diseases, with many subclassifications. Due to limited time and resources, this study does not contain a comprehensive analysis. Nevertheless, cause-of-death reports suggest that these diseases are associated with the highest mortality rates, which urgently need to be studied. It is necessary to promote multidisciplinary collaboration in basic and clinical research on cardiovascular diseases and tumors to establish appropriate prevention and treatment strategies in the future.

Research on mortality can provide valuable information that could aid in the assessment of the efficacy of health systems and policies, as well as identify areas and resources for immediate and future action. In summary, malignant tumors and cerebrovascular diseases showed overall downward trends, suggesting that China has made progress in the prevention and control of NCDs to a certain extent. The mortality rates of the three diseases were higher among males than females and higher in rural areas than urban areas. The equalization of basic public health services needs to be further strengthened, and health equity needs to be improved. Rural females aged over 65 years are at higher risk of heart disease, indicating that health education should include basic health knowledge, self-care techniques, and first aid skills. Additionally, policy-makers should dedicate more attention to promoting the health of the aging population in all aspects.

## Data Availability

The crude mortality data are derived from the China Health Statistical Yearbook at https://data.cnki.net/yearbook/Single/N2021020144.
