# Supplementary material for: Trend Analysis of the Mortality Rates of the Top Three Causes of Death Among Chinese Residents from 2003 to 2019
Source: Int J Public Health. 2022 Sep 6;67:1604988. doi: 10.3389/ijph.2022.1604988 (PMC9485456; doi:10.3389/ijph.2022.1604988)
Supplement: Supplementary file 1 [file DataSheet1.PDF]

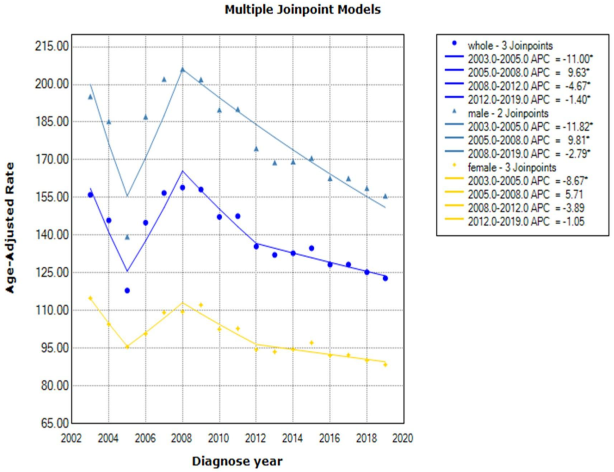

a） malignant tumors

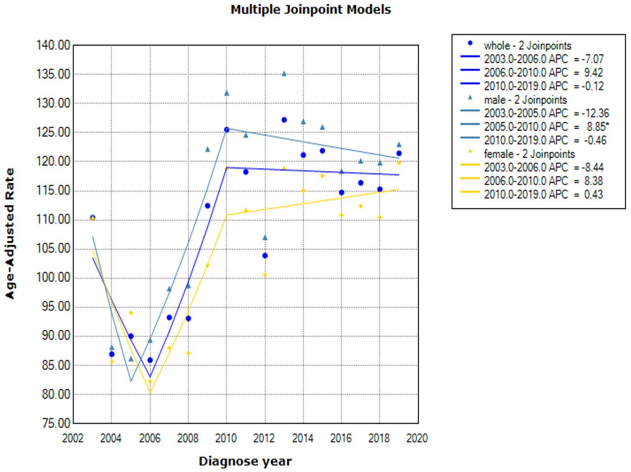

b） heart disease

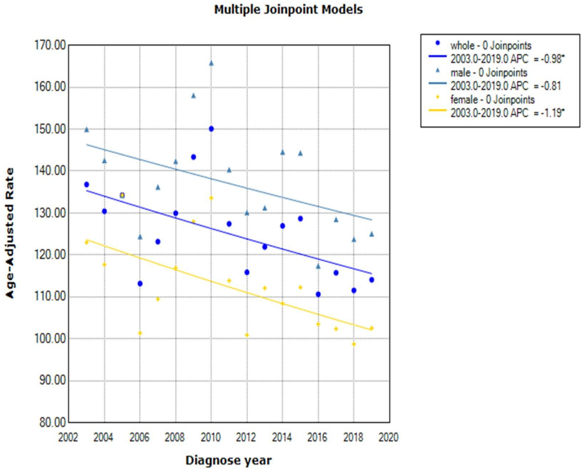

c） cerebrovascular disease

Figure S1 **Mortality trends of the three diseases by sex**（China, 2022）

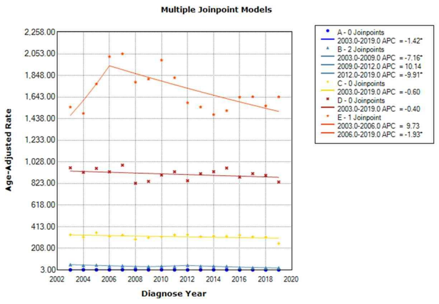

malignant tumors a) Male/urban

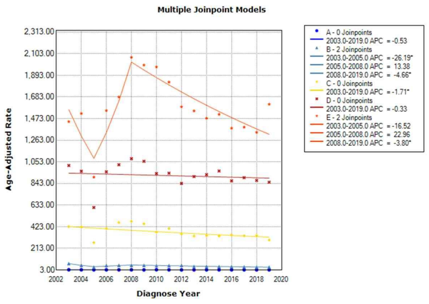

b) Male/rural

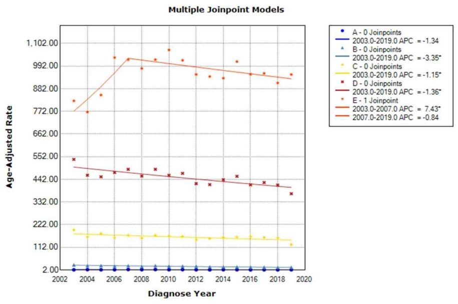

c) Female/urban

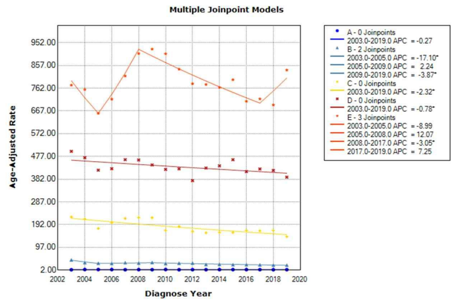

d) Female/rural

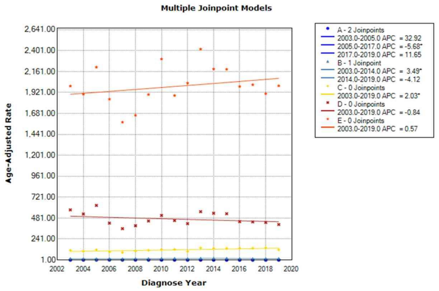

heart disease a) Male/urban

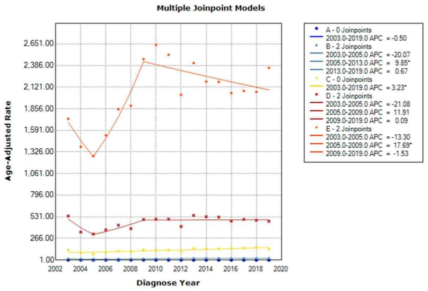

b) Male/rural

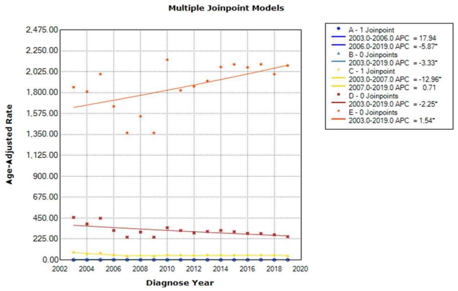

c) Female/urban

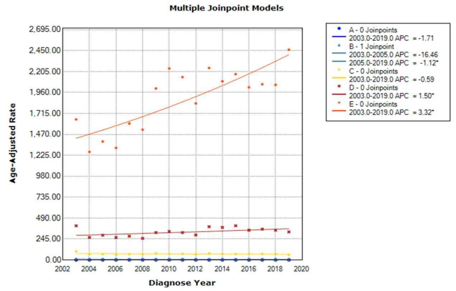

d) Female/rural

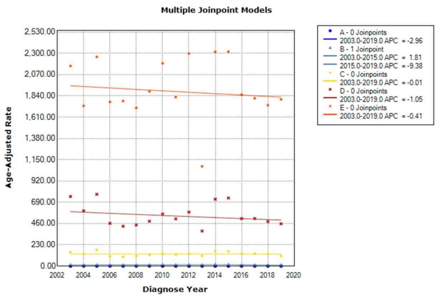

cerebrovascular disease a) Male/urban

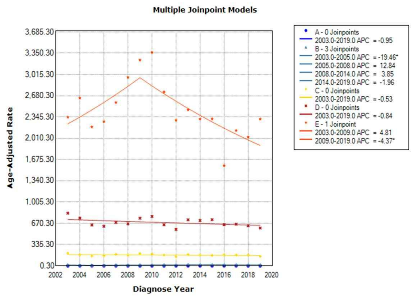

b) Male/rural

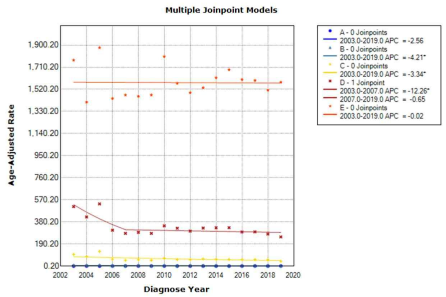

c) Female/urban

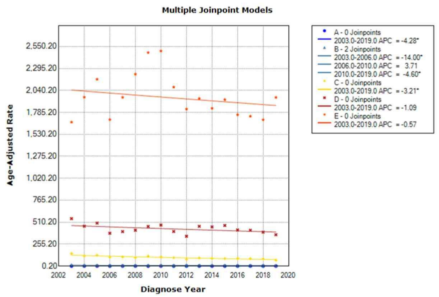

d) Female/rural

Notes: **A** 0-19 **B** 20-49 **C** 50-64 **D** 65-74 **E** ≥75

Figure S2 Mortality trends of the three diseases by sex, region and age group （China， 2022）
